# Compliance Toward Protective Precautions During and After the Lockdown Among Citizens of Riyadh

**DOI:** 10.7759/cureus.20320

**Published:** 2021-12-10

**Authors:** Wajdan Alassaf, Shuruq AlQahtani, Talah Binladen, Sarah Almagushi, Hind Alsaeed, Ghadah Alhussein, Lama Albakr, Waad Alshahrani

**Affiliations:** 1 Emergency Department, Princess Nourah Bint Abdulrahman University, Riyadh, SAU; 2 Emergency Medicine, King Abdullah Bin Abdulaziz University Hospital, Riyadh, SAU; 3 College of Medicine, Princess Nourah Bint Abdulrahman University, Riyadh, SAU

**Keywords:** pandemic fatigue, covid-19, personal protective precautions, vaccination, compliance

## Abstract

Objective: The emergence of the novel coronavirus disease 2019 (COVID-19) has resulted in precautionary measures by governments worldwide to contain the spread of the virus. However, the degree of compliance toward personal precautionary measures has varied, despite the urgent need to contain the pandemic. This study aimed to assess the level of compliance toward personal protective precautions during and after the lockdown among residents of Riyadh, Saudi Arabia.

Methods: We conducted a cross-sectional study with 1,108 residents using an online survey to collect sociodemographic data, compliance with personal protective measures, and other factors during and after the lockdown. Statistical Package for Social Sciences (SPSS) version 26 (IBM SPSS Statistics, Armonk, NY) was used for data analysis.

Results: During the lockdown, 35.5% of respondents were “very compliant,” whereas 30.1% were “less compliant.” Additionally, compliance levels in those who depended on official sites as their primary source of information were significantly higher than those who depended on their family, friends, and social media. Approximately 39.76% of those who were “very compliant” intended on getting vaccinated against COVID-19, while 28% of those with high compliance did not intend on getting vaccinated. Further, compliance levels were high in participants who were not in direct contact with COVID-19 cases as well.

Conclusion: This study highlights the importance of maintaining the level of compliance toward personal precautionary measures against COVID-19 even after lockdown release to prevent further waves. This study showed that compliance toward personal protective measures was high for those who did not have direct contact with COVID-19 positive people after lockdown, those planning for inoculation, and those insuring applications of precautionary measures by their children. Additionally, higher compliance was noted in participants who sought information from official sites.

## Introduction

In the last two decades, several pandemics have occurred, including the 2009 H1N1 virus [1]. On March 11, 2020, the World Health Organization (WHO) declared coronavirus disease 2019 (COVID-19) a pandemic [2]. On October 22, 2020, 41 million COVID-19 cases were reported globally, with 1.31 million deaths. Simultaneously, 343,000 COVID-19 cases and 5,235 deaths were reported in the Kingdom of Saudi Arabia [3]. Saudi Arabia implemented drastic measures to contain the pandemic including awareness promotion, hospital preparation, and multiple stages of lockdown [4]. Therefore, it is essential to evaluate the effectiveness of precautionary measures on the level of compliance toward them during and after lockdown. Both government and individual efforts influence awareness and compliance toward personal protective precautions in the general public to fight against infectious diseases. Moreover, stringent government actions to reduce the spread of COVID-19 are essential in containing the spread of infection [5], as prior studies report that quarantine of infected and suspected cases and travel suspension to and from areas of the outbreak were accepted and agreed upon by 95% of Arabs [6]. Additionally, early intervention policies implemented in Saudi Arabia such as suspension of schools, universities, Umrah (i.e., Islamic pilgrimage), praying in mosques, conferences, and all types of gatherings facilitated governmental control over reported and non-reported cases of infection [7]. Moreover, lockdown and suspension of all outdoor activities helped gauge a more comprehensive view of the gravity of COVID-19, thereby ensuring better community-level compliance [8]. Furthermore, increased awareness and compliance were also observed during the lockdown due to adequate access to social media and COVID-19-related news and information published by the Ministry of Health (MoH) in Saudi Arabia and other healthcare facilities. However, studies reveal a bidirectional relationship between long-term public compliance and psychological symptoms during lockdowns, including anxiety, stress, and depression [8,9]. Based on a study conducted in the United Kingdom, while people were more compliant at the beginning of the lockdown, with time, they developed “lockdown fatigue,” which reduced their compliance toward precautionary measures [10]. Numerous studies have observed lowered compliance toward protective precautions against COVID-19 after the lockdown was lifted. For instance, a study showed that while China's factory workers reported wearing face masks, maintaining hand hygiene, and avoiding social gatherings, they did not avoid crowded places [11,12]. However, as global quarantine measures had a significant psychological impact on people, these experiences may have led to an increase in protective precautions such as frequent hand washing, avoiding crowded places, and wearing face masks even after the lockdown release. Thus, after lockdown, the increase in compliance toward protective measures may be related to the fear of another mandatory lockdown and consequences of financial and work-related issues [13].

Prior studies report that sociodemographic factors influence the practice of personal precautionary measures. For instance, a study revealed that people above 34 years were more compliant compared with those who were younger; moreover, married people showed better protective precautions practices [14]. Studies have also revealed that a higher educational level was associated with better knowledge and practice of precautionary measures against COVID-19 [2,14]. However, males were reported to be less compliant than females in multiple studies [2,14]. Additionally, high socioeconomic status played a significant role in the practice of precautionary measures [3]. While various studies have examined compliance toward COVID-19 precautionary measures, no study has compared compliance levels during and after the lockdown in Saudi Arabia.

In our study, we aimed to assess the compliance levels of citizens of Riyadh, Saudi Arabia, toward precautionary measures during and after lockdown, in addition to their basic knowledge about COVID-19. We hypothesized that lockdown measures would increase their sense of seriousness and hence their level of compliance toward protective measures. Additionally, those with appropriate knowledge about the disease and precautionary measures would maintain their compliance level even after the lockdown is lifted.

## Materials and methods

Study design

We conducted a cross-sectional study with residents of Riyadh, Saudi Arabia, by administering an online survey immediately after the lockdown was lifted.

Recruitment procedure

Using non-probability convenience sampling, the survey was conducted electronically through social media and message-based communication applications where a short paragraph about the study alongside a link to the online study platform was sent to residents aged 18 years and above who volunteered to participate in the study (the first page of the study mentioned that if you agree to participate please precede with answering the questionnaire). The G power analysis program with a 3% margin of error and 97% confidence level estimated a minimal sample size of 1,000; we collected data from 1,108 respondents.

Measures

The questionnaire was designed using Research Electronic Data Capture (REDCap). We initially used the English version of the questionnaire, which was later translated to Arabic. We piloted the questionnaire on 10 people from the general public to ensure its clarity and readiness for publication. The questionnaire was reviewed by experts who supervised the translation process. The link was shared on social media applications, including WhatsApp, Instagram, and Twitter, during the COVID-19 pandemic but after the lockdown period in Saudi Arabia (i.e., January to February 2021). The questionnaire consisted of two sections with 45 questions. The first section comprised participants' sociodemographic data such as gender, age, marital status, educational level, work status, and socioeconomic status. Socioeconomic status was divided into four sections: “insufficient and borrows money,” “insufficient,” “sufficient,” i.e., for basics, and “more than sufficient,” i.e., enough for basics and luxuries. The second section focused on compliance toward personal protective measures (hand hygiene, face touching, safe distance, and other factors during and after the lockdown). Using a scoring system, the level of compliance was classified into “very compliant,” “compliant,” or “less compliant” according to participants’ scores through a Likert scale ranging from 1 to 5.

Data analysis

Statistical Package for Social Sciences (SPSS) version 26 (IBM SPSS Statistics, Armonk, NY) was used for data analysis; we described variables using means, standard deviations (SD), or percentages, as deemed appropriate. Furthermore, the chi-square test was used to determine the association between the quantitative variables. A p-value less than 0.05 was considered statistically significant.

Ethical issues

Written informed consent was obtained from all the participants prior to their participation. The study was approved by the Institutional Review Board (IRB) of Princess Nourah Bint Abdulrahman University (IRB log number: 20-0471; December 28, 2020).

## Results

Participants’ sociodemographic characteristics

Table 1 shows that a total of 1,108 respondents completed the survey. The mean age of the sample was 34 years, and females accounted for 85.7% of the respondents. Residents of Riyadh accounted for 80.3% of the sample. Moreover, a majority of the participants (72.1%) held a bachelor's degree, while approximately 40% were employed, and one-third of the sample included students (30.9%). A majority of the participants were non-healthcare workers (approximately 94%). Approximately 88.7% reported that they had been infected with COVID-19, while 33.5% of them were in direct contact with an individual who was tested positive for COVID-19.

**Table 1:** Participants’ sociodemographic characteristics. * Values are presented as mean ± standard deviation or number (%). HTN, hypertension; COPD, chronic obstructive pulmonary disease; COVID-19, coronavirus disease 2019.

Statistics	Item
34.1 ± 12.3	Age
Gender	
950 (85.7%)	Females
Education level	
161 (16.8%)	High school
799 (72.1%)	Bachelor's
118 (10.6%)	Postgraduate
Marital status	
514 (46.4%)	Single
594 (53.6%)	Married
Live in Riyadh	
445 (80.3%)	Yes
Work sector	
321 (72.1%)	Public
118 (26.5%)	Private
Work status	
445 (40.2%)	Employed
88 (7.9%)	Unemployed
342 (30.9%)	Student
233 (21.0%)	Homemaker
Healthcare workers	
65 (5.9%)	Yes
Income	
725 (65.4%)	More than sufficient
313 (28.2%)	Sufficient
41 (3.7%)	Insufficient
29 (2.6%)	Insufficient and borrows money
Chronic diseases	
78 (7.0%)	Diabetes
51 (4.6%)	HTN
1 (0.1%)	COPD
86 (7.8%)	Asthma
92 (7.8%)	Others
850 (76.7%)	No chronic disease
Infected with COVID-19	
983 (88.7%)	Yes
Direct contact with COVID-19	
371 (33.5%)	Yes

Participants’ level of compliance toward personal protective measures during the lockdown

Table 2 shows that with regard to age, the most compliant age group toward the personal protective measures was between 51 and 61 years, with 39.5% and 38.4% being compliant and very compliant, respectively. This was followed by the 29-39 age group, with 36.9% and 37.9% being compliant and very compliant, respectively. However, the least compliant age group was 18-28 years, as 34.0% of respondents from this age group showed poor compliance. Moreover, a higher proportion of females, compared with males, were very compliant, with a difference of 7.1% between the two groups; however, this difference was not significant (p > 0.05). The results also showed that 37.5% of single participants and 33.7% of married participants were very compliant. In terms of educational level, 60% of those with below high school education and 38.0% of those with high school education were very compliant toward personal protective measures. However, approximately one-third (34.2%) of those with postgraduate level education had poor compliance toward personal protective measures. Moreover, 45.7% of those with insufficient income were very compliant, whereas one-third (32.5%) of those with more than sufficient income were poorly compliant. Additionally, 70.2% of employees were compliant and very compliant, and 75.5% of healthcare workers were compliant and very compliant.

**Table 2:** Participants’ compliance level during the lockdown. COVID-19, coronavirus disease 2019.

		Less compliant (less than 75)	Compliant (75-85)	Very compliant (more than 85)
Age group	18-28	146 (34.0%)	135 (31.4%)	149 (34.7%)
	29-39	52 (%25.2)	78 (37.9%)	76 (36.9%)
	40-50	75 (29.9%)	87 (34.7%)	89 (35.5%)
	51-61	19 (22.1%)	34 (39.5%)	33 (38.4%)
	62-72	4 (33.3%)	5 (41.7%)	3 (25%)
Gender	Female	253 (30.0%)	282 (33.5%)	308 (36.5%)
Marital status	Married	156 (29.9%)	190 (36.4%)	176 (33.7%)
Education	Below high school	0 (0.0%)	3 (60.0%)	2 (40.0%)
	High school	45 (27.6%)	56 (34.4%)	62 (38.0%)
	Bachelor's	214 (30.3%)	241 (34.1%)	252 (35.6%)
	Postgraduate	38 (34.2%)	39 (35.1%)	34 (30.6%)
Place of work	Public sector	82 (27.8%)	112 (38.0%)	101 (34.2%)
	Private sector	36 (34.6%)	33 (31.7%)	35 (33.7%)
	Business	2 (50.0%)	1 (25.0%)	1 (25.0%)
Work status	Employed	120 (29.8%)	146 (36.2%)	137 (34.0%)
	Unemployed	23 (28.0%)	36 (43.9%)	23 (28.0%)
	Student	95 (30.8%)	94 (30.5%)	119 (38.6%)
	Homemaker	59 (30.6%)	63 (32.6%)	71 (36.8%)
Are you a health care worker?	Yes	14 (24.6%)	23 (40.4%)	20 (35.1%)
Do you live in Riyadh?	Yes	246 (31.1%)	276 (34.8%)	270 (34.1%)
Socioeconomic status	More than sufficient	210 (32.5%)	214 (33.1%)	223 (34.5%)
	Sufficient	72 (26.1%)	104 (37.7%)	100 (36.2%)
	Insufficient	7 (20.0%)	12 (34.3%)	16 (45.7%)
	Insufficient and borrows money	8 (28.6%)	9 (32.1%)	11 (39.3%)
Disease		55 (24.7%)	81 (36.3%)	87 (39%)
No disease		242 (31.7%)	258 (33.8%)	263 (34.5%)
Infected with COVID-19	Yes	262 (29.8%)	298 (33.9%)	319 (36.3%)
Infected with COVID-19	No	36 (33.3%)	41 (38.0%)	31 (28.7%)
Direct contact with individuals infected with COVID-19	Yes	108 (32.4%)	122 (36.6%)	103 (30.9%)
Do you intend on getting vaccinated against COVID-19?	Yes	99 (25.4%)	137 (35.1%)	154 (39.5%)
	No	52 (40.0%)	42 (32.3%)	36 (27.7%)
	Have not decided yet	146 (31.3%)	160 (34.3%)	160 (3)4.3%
What is your source of COVID-19-related information?	Family & friends	54 (39.1%)	39 (28.3%)	45 (32.6%)
	Social media	144 (37.1%)	128 (33%)	116 (29.9)
	Official sites	239 (27.9%)	301 (35.1%)	317 (37%)
If you are a parent or responsible for a child, to what extent do you ensure that your child is performing personal protective measures in public places?	0%	4 (66.7%)	2 (33.3%)	0 (0.0%)
	20%	12 (66.7%)	5 (27.8 %)	1 (5.6%)
	50%	41 (61.2%)	17 (25.4%)	9 (13.4%)
	75%	54 (30.2%)	77 (43.0%)	48 (26.8%)
	100%	51 (18.7%)	88 (32.2%)	134 (49.1%)
	Not responsible for a child	135 (30.5%)	150 (33.9%)	158 (35.7%)

**Figure 1:**
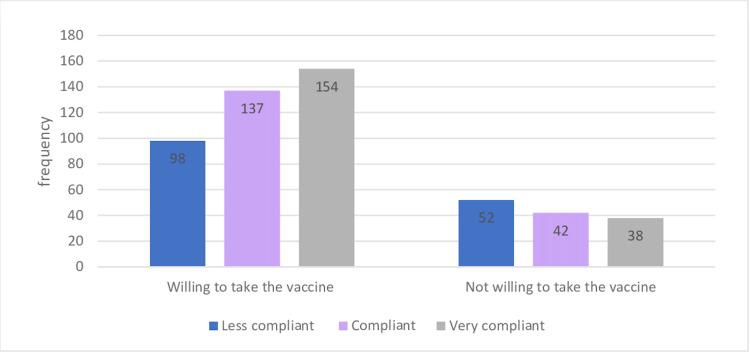
Association between the level of compliance and intention for inoculation during the lockdown.

In terms of intention for inoculation and compliance level, 39.5% of those who intended on getting vaccinated against COVID-19 were very compliant, whereas only 27.7% who did not intend on getting vaccinated were very compliant (p = 0.02) (Figure 1). While analyzing the association between the level of compliance and the source of COVID-19-related information, only 13.89% of the respondents depended on family and friends as their primary source of information, whereas official sites were the preferred source (86.91%) of information among respondents. Interestingly, during the lockdown, those who depended on family and friends as their source of knowledge secured the lowest compliance score (39.1%). Moreover, 37% of respondents were very compliant during the lockdown (p = 0.04).

**Figure 2:**
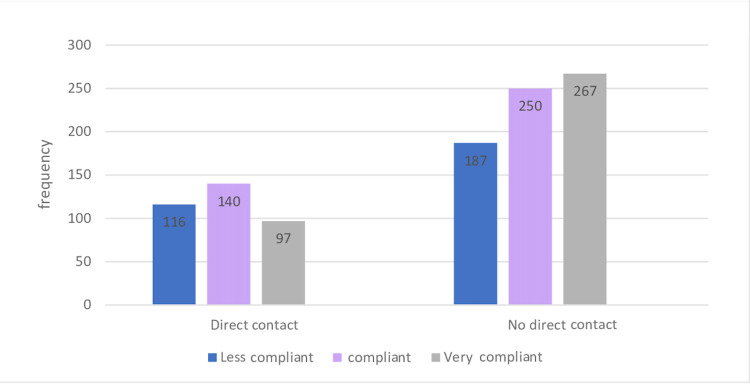
Association between the level of compliance and direct contact with COVID-19 infected individuals during the lockdown. COVID-19, coronavirus disease 2019.

Regarding the relationship between COVID-19 infection and level of compliance, 36.3% of those who had COVID-19 were very compliant, whereas 28.7% of those who were non-infected were very compliant. The level of compliance during lockdown was higher in those who did not have contact with COVID-19 positive cases (Figure 2). Additionally, 49.8% of those who always ensured that their children practiced personal protective measures were very compliant, while 80% of those who did not keep a check on their children’s precautionary measures were poorly compliant (p < 0.001).

Participants’ level of compliance toward personal protective measures after lockdown release

**Figure 3:**
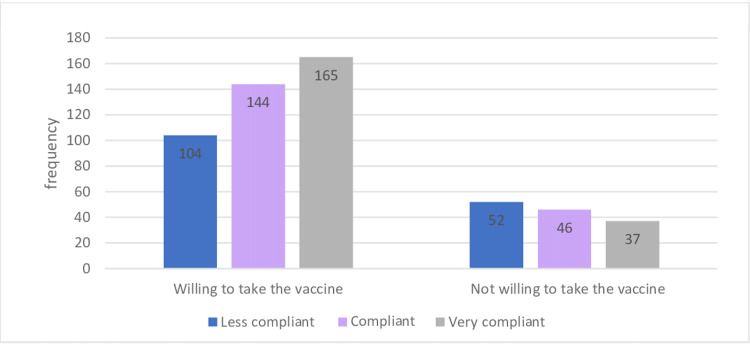
Association between the level of compliance and intention for inoculation after lockdown.

**Figure 4:**
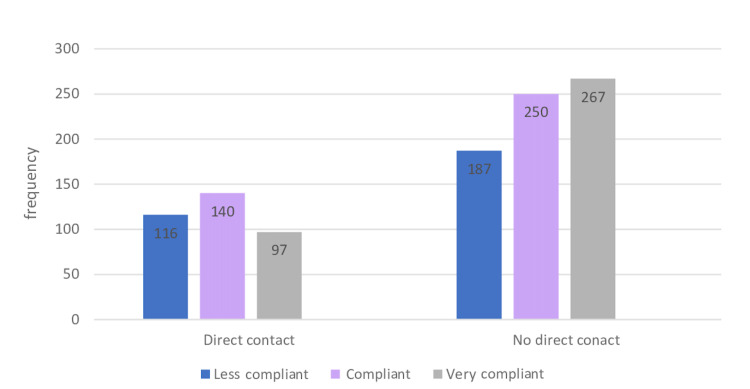
Association between the level of compliance and direct contact with COVID-19 infected individuals. COVID-19, coronavirus disease 2019.

Table 3 shows that after the lockdown, the 51-61 age group was the most compliant, with 40.9% being compliant and 38.7% being very compliant. However, nearly one-third (32%) of those in the 18-28 age group were poorly compliant. A higher proportion of females were very compliant, compared with males, with a difference of 7.5% between the two groups. Moreover, the majority (80%) of those with below high school educational level were compliant, whereas 38.4% of those with postgraduate level education were poorly compliant; however, no significant association between educational level and compliance (p = 0.104) was found. Moreover, 36.7% of residents of Riyadh were compliant. With respect to the COVID-19 inoculation intention, 40% of those who intended on getting vaccinated against COVID-19 were very compliant (Figure 3). In comparison, 27.4% of those who did not intend on getting inoculated were very compliant (p = 0.006). Furthermore, 38.3% of those with chronic diseases were very compliant, compared to 33.5% healthy participants. Regarding the source of COVID-19-related information and compliance level, more than one-third (37.6%) of those who depended on social media as their source of COVID-19-related information were classified as less compliant, whereas 36.8% of those who depended on official sites were very compliant (p = 0.016). Almost one-third (35.8%) of non-infected participants presented poor compliance. However, in comparison, 27.8% of the infected participants were poorly compliant. Additionally, 39.7% of those who came in direct contact with an individual who tested positive for COVID-19 were compliant; moreover, a significant positive correlation was found between direct contact with an individual who had COVID-19 and compliance toward precautionary measures (p = 0.003) (Figure 4).

**Table 3:** Participants’ compliance levels after lockdown release. COVID-19, coronavirus disease 2019.

		Less compliant (less than 75)	Compliant (75-85)	More compliant (more than 85)	p-value
Age group	18-28	144 (32.0%)	156 (34.7%)	150 (33.3%)	0.404
	29-39	57 (25.1%)	94 (41.4%)	76 (33.5%)	
	40-50	78 (28.6%)	98 (35.9%)	97 (35.5%)	
	51-61	19 (20.4%)	38 (40.9%)	36 (38.7%)	
	62-72	4 (30.8%)	4 (30.8%)	5 (38.5%)	
Gender	Male	46 (30.7%)	62 (41.3%)	42 (28.0%)	0.194
Marital status	Married	156 (27.5%)	221 (39.0%)	190 (33.5%)	0.316
Education	Below high school	0 (0.0%)	4 (80.0%)	1 (20.0%)	0.104
	High school	49 (27.4%)	63 (35.2%)	67 (37.4%)	
	Bachelor’s	211 (27.7%)	287 (37.7%)	263 (34.6%)	
	Postgraduate	43 (38.4%)	36 (32.1%)	33 (29.5%)	
Place of work	Public sector	85 (27.2%)	120 (38.3%)	108 (34.5%)	0.662
	Private sector	36 (32.4%)	45 (40.5%)	30 (27.0%)	
	Business	2 (33.3%)	2 (33.3%)	2 (33.3%)	
Work status	Employed	123 (28.6%)	167 (38.8%)	140 (32.6%)	0.723
	Unemployed	25 (29.4%)	35 (41.2%)	25 (29.4%)	
	Student	95 (29.1%)	111 (34.0%)	120 (36.8%)	
	Homemaker	60 (27.8%)	77 (35.6%)	79 (36.6%)	
Are you a health care worker?	Yes	18 (29.0%)	20 (32.3%)	24 (38.7%)	0.692
Do you live in Riyadh?	Yes	255 (30.0%)	312 (36.7%)	283 (33.3%)	0.112
Socioeconomic status	More than sufficient	208 (30.2%)	246 (35.7%)	235 (34.1%)	0.809
	Sufficient	80 (26.5%)	117 (38.7%)	105 (34.8%)	
	Insufficient	8 (21.6%)	16 (43.2%)	13 (35.1%)	
	Insufficient and borrows money	7 (24.1%)	11 (37.9%)	11 (37.9%)	
Disease		60 (24.2%)	93 (37.5%)	95 (38.3%)	0.157
No disease		243 (30%)	297 (36.7%)	269 (33.5%)	0.146
Infected with COVID-19	Yes	261 (27.8%)	344 (36.7%)	333 (35.5%)	0.086
Infected with COVID-19	No	43 (35.8%)	46 (38.3%)	31 (25.8%)	0.067
Direct contact with individuals infected with COVID-19	Yes	116 (32.9%)	140 (39.7%)	97 (27.5%)	0.003
What is your source of COVID-19-related information?	Family & friends	53 (36.6%)	56 (38.6%)	36 (24.8%)	0.016
	Social media	157 (37.6%)	151 (36.2%)	109 (26.1%)	
	Official sites	229 (25.1%)	347 (38%)	336 (36.8%)	
If you are a parent or responsible for a child, to what extent do you ensure that your child is performing personal protective measures in public places?	0%	4 (80.0%)	1 (20.0%)	0 (0.0%)	
	20%	12 (60.0%)	6 (30.0%)	2 (10.0%)	
	50%	43 (60.6%	17 (23.9%)	11 (15.5%)	
	75%	52 (28.7%)	87 (48.1%)	42 (23.2%)	
	100%	43 (15.4%)	97 (34.8%)	139 (49.8%)	
	Not responsible for a child	133 (29.6%)	167 (37.1%)	150 (33.3%)	

Comparison between participants’ compliance levels during and after the lockdown

Table 4 shows that after the lockdown release, no age group showed any change in their compliance during the lockdown and after the lockdown was lifted. From a gender perspective, males showed increased compliance after lockdown, 6% more than females. Approximately one-third (35.2%) of married participants had better compliance after the lockdown release. Moreover, 60% of those with below high school educational level and 41.9% of those with high school level education had decreased compliance after lockdown release; however, there was no significant association between educational level and compliance after lockdown (p = 0.104). Moreover, half (50%) of those with insufficient financial resources and debts presented no change in their compliance after the lockdown was lifted. Over one-third (36.1%) of residents of Riyadh demonstrated a reduction in their compliance level after lockdown release. Additionally, 32.6% and 33.9% of employees and students, respectively, showed a decrease in their compliance level, whereas 39.3% of healthcare workers demonstrated no change in their compliance after lockdown release.

**Table 4:** Comparison between participants during the lockdown and after lockdown release. COVID-19, coronavirus disease 2019.

		No change	Increased after lockdown release	Decreased after lockdown release	p-value
Age group	18-28	189 (41.4%)	111 (24.3%)	156 (34.2%)	0.816
	29-39	82 (37.1%)	61 (27.6%)	78 (35.3%)	
	40-50	101 (38.7%)	68 (26.1%)	92 (35.2%)	
	51-61	43 (48.9%)	18 (20.5%)	27 (30.7%)	
	62-72	5 (41.7%)	3 (25%)	4 (33.3%)	
Gender	Male	63 (40.9%)	46 (29.9%)	45 (29.2%)	0.229
Marital status	Married	210 (38.3%)	146 (26.6%)	193 (35.2%)	0.227
Work status	Employed	176 (41.6%)	109 (25.8%)	138 (32.6%)	0.754
	Unemployed	36 (42.9%)	20 (23.8%)	28 (33.3%)	
	Student	137 (41.5%)	81 (24.5%)	112 (33.9%)	
	Homemaker	72 (35.5%)	52 (25.6%)	79 (38.9%)	
Socioeconomic status	More than sufficient	278 (40.4%)	178 (25.9%)	232 (33.7%)	0.631
	Sufficient	113 (39.4%)	73 (25.4%)	101 (35.2%)	
	Insufficient	16 (43.2%)	5 (13.5%)	16 (43.2%)	
	Insufficient and borrows money	14 (50%)	6 (21.4%)	8 (28.6%)	
Education	Below high school	1 (20%)	1 (20%)	3 (60%)	
	High school	58 (33.7%)	42 (24.4%)	72 (41.9%)	0.22
	Bachelor’s	316 (42.3%)	191 (25.6%)	240 (32.1%)	
	Postgraduate	46 (39.7%)	28 (24.1%)	42 (36.2%)	
Place of work	Public sector	124 (40.5%)	81 (26.5%)	101 (33%)	0.743
	Private sector	51 (45.5%)	26 (23.2%)	35 (31.3%)	
	Business	1 (20%)	2 (40%)	2 (40%)	
Are you a healthcare worker?	Yes	24 (39.3%)	17 (27.9%)	20 (32.8%)	0.883
Do you live in Riyadh?	Yes	332 (39.7%)	203 (24.3%)	301 (36%)	0.064
Infected with COVID-19	Yes	376 (40.5%)	241 (26%)	311 (33.5%)	0.155
Infected with COVID-19	No	45 (39.8%)	22 (19.5%)	46 (40.7%)	0.205
Direct contact with individuals infected with COVID-19	Yes	153 (43.6%)	77 (21.9%)	121 (34.5%)	0.174
Disease		110 (45.8%)	56 (23.3%)	74 (30.8%)	0.154
No diseases		311 (38.9%)	206 (25.8%)	283 (35.4%)	0.185
Do you intend on getting vaccinated against COVID-19?	Yes	175 (42.2%)	102 (24.6%)	138 (33.3%)	0.77
	No	51 (37.8%)	39 (28.9%)	45 (33.3%)	
	Have not decided yet	195 (39.8%)	121 (24.7%)	174 (35.5%)	
What is your source of COVID-19-related information?	Family & friends	70 (47.3%)	27 (18.2%)	51 (34.5%)	0.071
	Social media	159 (38.1%)	98 (23.5%)	160 (38.4%)	
	Official sites	379 (42.1%)	233 (25.9%)	289 (32.1%)	
If you are a parent or responsible for a child, to what extent do you ensure that your child is performing personal protective measures in public places?	0%	2 (33.3%)	2 (33.3%)	2 (33.3%)	0.004
	20%	5 (25.0%)	8 (40.0%)	7 (35.0%)	
	50%	16 (22.5%)	22 (31.0%)	33 (46.5%)	
	75%	65 (35.3%)	40 (21.7%)	79 (42.9%)	
	100%	124 (43.4%)	80 (28.0%)	82 (28.7%)	
	Not responsible for a child	209 (44.2%)	110 (23.3%)	154 (32.5%)	

Moreover, 35.4% of healthy participants showed decreased compliance post lockdown, while 45.8% of those with chronic diseases demonstrated no change in their compliance toward personal protective measures. Additionally, the highest percentage (25.9%) of positive change in compliance was found among those who depended on official sites for COVID-19-related information. Regarding COVID-19 infection and the level of compliance, 40.5% of the participants who got infected with COVID-19 showed no change in their compliance level during and after lockdown, while 40.7% of those who did not get infected with COVID-19 showed a reduction in their compliance level after lockdown release.

Furthermore, 43.4% of the participants who always ensured that their children performed personal protective measures demonstrated no change in their compliance after lockdown release. However, those who kept a check on their children’s personal protective measures only sometimes showed a reduction in their compliance level after lockdown release (p = 0.004).

## Discussion

The most compliant group during the lockdown were older adult participants. This result can be attributed to increased comorbidities that result in compromised immunity among older people, thus, making them more cautious regarding infections. A study by Almutairi et al. [14] supported this finding, as they found that older participants were significantly and directly associated with higher compliance to precautionary measures. Moreover, the results of this study are consistent with a prior study [15], which revealed that women were more caring toward their family and friends, thus, ensuring their compliance with personal protective measures, while men were more concerned about the impact of the pandemic on the economy and society. Thus, it was not surprising to observe that a high percentage of employees had increased compliance, as they were at higher risk of getting infected at their workplace.

A prior study found that persons who intend on getting vaccinated are likely to be more knowledgeable about the impact of COVID-19 on health and the benefits of the vaccine, and thus, are more compliant. This explanation is supported by a study in China in 2020 [11], which found that family and friends were most depended on as sources of pandemic-related information, which can explain the extent of misinformation spread by family and friends who do not rely on official sources, such as WHO and MoH. During the period after lockdown, similar to older people, participants with chronic diseases have compromised immunity, thus, making them more susceptible to COVID-19. Participants who relied on social media had poor compliance, as they referred to unofficial information, which does not urge compliance toward personal protective measures. Additionally, such participants are more likely to spread misinformation. This finding is supported by Seo’s [16] study, which revealed that those who spent more time referring to information about COVID-19 through government’s official sites had better knowledge and understanding of the pandemic situation; thus, elevated compliance levels toward personal protective measures were observed in these groups. Similarly, the results of our study showed that non-infected participants showed poor compliance, which might be because people who did not get infected may have a sense of false immunity; therefore, they act carelessly, decreasing their compliance. Surprisingly, a study conducted in China contradicted our findings where 99.2% of those who were COVID-19 positive were compliant with four personal nonpharmaceutical interventions, such as handwashing, proper coughing habits, social distancing, and mask-wearing [17].

Interestingly, a high percentage of participants below high school educational level demonstrated decreased compliance level after lockdown release. Thus, our study demonstrated the crucial role of socioeconomic status in compliance. A high percentage of those with insufficient financial resources and debts demonstrated no change in their compliance level. However, this study does have a few limitations. Data were collected using an online REDCap form, which was distributed through a particular group of people rather than all demographic categories such as the elderly and children. Thus, the results of this study may not be generalizable to the entire population, as it lacks a large and randomly distributed representative sample. However, despite these limitations, the findings of this study highlight the importance of maintaining the level of compliance toward personal precautionary measures against COVID-19 even after lockdown release to prevent further waves.

## Conclusions

Previous studies show that sociodemographic factors including age, educational level, gender, and socioeconomic status influence the practice of precautionary measures. This study showed that compliance toward personal protective measures was very high for those who did not have direct contact with COVID-19 positive people after lockdown. Additionally, compliance was very high during and after lockdown for people with the intention for inoculation. Furthermore, parents who ensured that their children practiced precautionary measures showed high compliance during and after lockdown release, with an increase in their compliance after the release of the lockdown. Moreover, people who depended on their family, friends, and social media as sources of COVID-19-related information reported low compliance, compared with those who sought information from official sites.

Finally, this study has several recommendations. The level of compliance regarding personal precautionary measures against COVID-19 should be maintained after the lockdown as the pandemic is still ongoing. This is also to prevent further waves. The results of this study emphasize the importance of adherence to precautionary measures during and after lockdown, which should be explained to the public in social media, schools, and newspapers to reach all populations regardless of their age, class, and educational level.
